# Screening for glucose-6-phosphate dehydrogenase deficiency in neonates: a comparison between cord and peripheral blood samples

**DOI:** 10.1186/s12887-017-0912-y

**Published:** 2017-07-11

**Authors:** Saif AlSaif, Ma. Bella Ponferrada, Khalid AlKhairy, Khalil AlTawil, Adel Sallam, Ibrahim Ahmed, Mohammed Khawaji, Khalid AlHathlol, Beverly Baylon, Ahmed AlSuhaibani, Mohammed AlBalwi

**Affiliations:** 1 Division of Neonatology, Department of Pediatrics, King Abdulaziz Medical City, Ministry of National Guard Health Affairs, P.O. Box 22490, Riyadh, 11426 Kingdom of Saudi Arabia; 2 Division of Neonatology, Department of Pediatrics, Prince Mohammad bin Abdulaziz Hospital, Ministry of National Guard Health Affairs, P.O. Box 40740, Al Madinah Al Medina, 41511 Kingdom of Saudi Arabia; 3 Division of Neonatology, Department of Pediatrics, King Abdulaziz Medical City, Ministry of National Guard Health Affairs, P.O. Box 9515, Jeddah, 21423 Kingdom of Saudi Arabia; 4 Department of Pathology and Laboratory Medicine, King Abdulaziz Medical City, Ministry of National Guard Health Affairs, P.O. Box 22490, Riyadh, 11426 Kingdom of Saudi Arabia; 5 Medical Genomics Research Department, King Abdullah International Medical Research Center, Ministry of National Guard Health Affairs, P.O. Box 22490, Riyadh, 11426 Kingdom of Saudi Arabia; 6 College of Medicine, King Saud bin Abdulaziz University for Health Sciences, P.O. Box 3660, Riyadh, 11481 Kingdom of Saudi Arabia

**Keywords:** Glucose-6-phosphate dehydrogenase deficiency, Screening, Neonates, Cord blood

## Abstract

**Background:**

The use of cord blood in the neonatal screening for glucose-6-phosphate dehydrogenase (G6PD) deficiency is being done with increasing frequency but has yet to be adequately evaluated against the use of peripheral blood sample which is usually employed for confirmation. We sought to determine the incidence and gender distribution of G6PD deficiency, and compare the results of cord against peripheral blood in identifying G6PD DEFICIENCY neonates using quantitative enzyme activity assay.

**Methods:**

We carried out a retrospective and cross-sectional study employing review of primary hospital data of neonates born in a tertiary care center from January to December 2008.

**Results:**

Among the 8139 neonates with cord blood G6PD assays, an overall incidence of 2% for G6PD deficiency was computed. 79% of these were males and 21% were females with significantly more deficient males (*p* < .001). Gender-specific incidence was 3.06% for males and 0.85% for females. A subgroup analysis comparing cord and peripheral blood samples (*n* = 1253) showed a significantly higher mean G6PD value for peripheral than cord blood (15.12 ± 4.52 U/g and 14.52 ± 4.43 U/g, respectively, *p* = 0.0008). However, the proportion of G6PD deficient neonates did not significantly differ in the two groups (*p* = 0.79). Sensitivity of cord blood in screening for G6PD deficiency, using peripheral G6PD assay as a gold standard was 98.6% with a NPV of 99.5%.

**Conclusion:**

There was no difference between cord and peripheral blood samples in discriminating between G6PD deficient and non-deficient neonates. A significantly higher mean peripheral G6PD assay reinforces the use of cord blood for neonatal screening since it has substantially low false negative results.

## Background

Despite the wide variability in the assessment methods for glucose-6-phosphate dehydrogenase deficiency, it remains the most prevalent enzyme deficiency in the world. It is estimated that nearly 330 million people may be affected by G6PD deficiency worldwide with a global prevalence of 4.9% [1]. G6PD deficiency in neonates is particularly important because it may have fatal consequences. It could cause severe neonatal jaundice, and if not recognized and managed early, it could lead to kernicterus with permanent neurologic sequelae, if not death [2, 3] In the Middle East, the prevalence estimates of G6PD deficiency is the second highest in the world at 6% (95% CI: 5.7–6.4, *p* < 0.001), the first being Sub Saharan Africa [1, 4]. For males alone, it is estimated to be 7.2% (95% CI: 6.6–7.7, *p* < 0.001). Local studies in Saudi Arabia have shown a wide variability in the region-specific prevalence. Riyadh has a prevalence of 2.0% to 3.8%, Qatif with 30.6%, and AlHasa with 14.7% [3, 5–7]. Most of these studies made use of the cord blood for screening in the neonates. The use of cord blood in the neonatal screening for metabolic diseases including G6PD deficiency is being done with increasing frequency in some centers [6, 8–12]. This method of specimen collection is convenient, easy, and more importantly it spares the neonate of unnecessary pain and stress. The premise is that; coming from the same individual, cord blood should reflect the same glucose-6-phosphate dehydrogenase (G6PD) levels as in the peripheral sample. This may not be entirely accurate; however, considering the tremendous physiologic changes in the newborn period that could affect G6PD activity. In this study, we sought to determine the incidence and gender distribution of G6PD deficiency among neonates using cord blood, and compared cord against peripheral blood in identifying G6PD deficiency in neonates using a quantitative enzyme activity assay.

## Methods

We carried out a retrospective, cross-sectional study employing review of primary hospital data of term and near term neonates born (>35 weeks of completed gestation) in a tertiary care center from January to December 2008. King Abdullah International Medical Research Center (KAIMRC), Ministry of National Guard Health Affairs (MNGHA), International Review Board (IRB) has approved this study protocol (RC09/106), and all patients were provided with written informed consent through their guardian/parent.

Cord blood is defined as a specimen collected from the umbilical artery at the time of delivery and peripheral blood is the blood obtained from any other site of the body within one week of age. Unless otherwise specified, we define G6PD deficiency as a G6PD quantitative assay by spectrophotometric analysis of ≤5.7 U/g Hb (unit of enzyme activity/g hemoglobin), as per laboratory recommendation.

Cord and venous blood samples were collected from each patient using conventional techniques into Vacutainer (BD Plymouth, PL6 7BP, U.K.) or Microtainer (Becton, Dickinson and Co., Franklin Lakes, NJ 07417, USA) tubes with K2EDTA as anticoagulant at a concentration of 1.8 mg/ml. Samples were accessioned into the Laboratory Information System (LIS), then their hemoglobin (Hb) levels were determined on same time and day as the G6PD analyses using Cell-Dyn Sapphire blood analyzers (Abbot Diagnostics Division, Abbot Park, IL 60064, USA). All samples were stored at 2-8C, batched and analyzed for G6PD enzymatic activity within 4–6 h of collection. Sadly, three infants died before the peripheral blood sample could be obtained for measurement of the G6PD.

One hundred microliters of well-mixed whole blood was pipetted in a test-tube containing 400 μl of a proprietary lysing reagent, mixed well and let stand for 5 min. An aliquot of this was poured into a sample cup using the only G6PD assay kit designed for both newborn and adults, which then was placed in the Udilipse Random Access Analyzer (United Diagnostics Industry, P.O. Box 9466, Dammam 31,413, Kingdom of Saudi Arabia). Once hemolysates were made, analysis was carried out immediately and strictly within an hour.

The principle of the test involves the catalysis of glucose-6-phosphate to 6-phosphogluconate by G6PD and reduction of NADP to NADPH in the following reaction [10] (**Glucose-6-phosphate + NADP ➔ 6-Phosphogluconate + NADPH + H** ^**+**^ **).**

The activity of G6PD was proportional to the rate of production of NADPH which possesses a peak Ultraviolet (UV) light absorption at 340 nm. Results from the Analyzer were automatically transmitted to LIS permitting access to patient’s previously estimated blood hemoglobin level, computed and reported results in units/g (of hemoglobin).

In carrying out the study, we collected the names and medical record numbers (MRN) of all newborns with G6PD quantitative assays (cord or peripheral) for the specified time interval (January to December 2008). This was the year that our institution began implementing universal G6PD deficiency neonatal screening. We then selected the neonates with cord G6PD assays and from this pool, the overall incidence and the gender distribution of G6PD deficiency was computed. However, due to the unavailability of G6PD molecular genotyping in our institute DNA analysis was not possible. There is a future plan to include DNA genotyping in a forthcoming study.

From among the newborns with cord blood G6PD assays, we picked out those who also had peripheral samples taken (presumably within one week of age). A subgroup analysis was carried out on this particular subset of patients (*n* = 1, 253) comparing the cord and peripheral G6PD values.

## Statistical analysis

Statistical analysis was performed using SPSS v.20 (IBM Corp., Armonk, NY, USA). The data were statistically tested by descriptive statistics. Kolmogrov-Smirnov test was used for normality and found that it was normally distributed. The Quantitative G6PD analysis and correlation between deficient and non-deficient groups for both cord and peripheral blood samples were performed using Student T-test and Pearson chi-square. *P*-value of <0.05 was considered statistically significant. Data was expressed as mean ± SD unless indicated otherwise (see Tables 1, 2 and 3).

**Table 1:** Subgroup analysis of G6PD Quantitative Assay for Neonates with both Cord and Peripheral blood samples

Description		Cord	Peripheral blood	*p*-value
G6PD Quantitative Assay (U/g)				0.0008*
Overall		14.52	15.12	
Mean		4.43	4.52	
Incidence of G6PD % at two cut-off values	5.7 U/g	5.83	5.59	0.796^a^
	8.05 U/g	7.98	7.18	0.450
Mean G6PD Quantitative Assay (U/g Hgb), at 5.7 U/g cut-off	Deficient	1.45 ± 1.16	1.64 ± 1.27	0.3711
		(*n* = 73)	(*n* = 70)	
	Non - Deficient	15.33 ± 3.09	15.92 ± 3.18	<0.001*
		(*n* = 1180)	(*n* = 1183)	
Mean G6PD Quantitative Assay (U/g Hgb), at 8.05 U/g cut-off	Deficient	2.95 ± 2.68	2.85 ± 2.56	0.8001
		(*n* = 100)	(*n* = 90)	
	Non - Deficient	15.52 ± 2.85	16.07 ± 3.01	<0.001*
		(*n* = 1153)	(*n* = 1163)	

*Level of significance: *p* = < 0.05

The cord results are statistically lower than the peripheral results *(p = 0.0008)*. This also holds true among the non-deficient subgroup of patients (*p < 0.001*) but NOT among the deficient neonates (*p = 0.3711 and p = 0.8001 using cut-off of 5.7 and 8.05 U/g Hb respectively*)

^a^Pearson chi^2^ test did not reveal any significant difference in the proportion of G6PD deficient neonates between cord and peripheral blood samples

**Table 2:** Gender distributed Differences in G6PD levels

Gender	Cut-off value	Cord		PB		*P*.value
		Mean	Range	Mean	Range	
Male	5.7 U/g	15.29 ± 3.28	(6–27)	15.78 ± 3.35	(5.8–30.5)	
Female		15.36 ± 2.90	(6–29)	16.02 ± 2.87	(5.7–27.7)	*P* < 0.001*
Male	8.5 U/g	15.45 ± 2.77	(8–27)	16.05 ± 2.7	(8.10–30.5)	
Female		15.57 ± 2.95	(8.10–29)	16.08 ± 3.12	(8.10–27.7)	*P* < 0.001*

*Level of significance: *p* = < 0.05, *Cord* Cord Blood, *PB* Peripheral Blood. Normal G6PD level distributed among different gender between the cord blood and peripheral blood samples

**Table 3:** Screening Indices for Cord Blood G6PD Assay at two cut-off values

	Cut-off values	
	5.7 U/g Hb	8.05 U/g Hb
Sensitivity	98.6%^a^	90%
	(CI:91.2, 99.9)	(CI:81.4, 95)
Specificity	99.7%	98.4%
	(CI:99.1%, 99.9%)	(CI:97.4%, 99%)
Positive Predictive Value	94.5%	81%
	(CI:85.8%, 98.2%)	(CI:71.7%, 87.9%)
Negative Predictive Value	99.5%	99.2%
	(CI:99.5%, 100%)	(CI:98.5%, 99.6%)
Accuracy	99.6%	97.77%

*CI* Confidence intervals

^a^98.6% of all the patients with truly deficient peripheral G6PD assays had deficient cord blood results with cut-off 5.7 U/g Hb once compared to 8.05 U/g Hb

## Results

There were a total of 8396 neonates admitted to the hospital between January and December 2008 whose G6PD assays were obtained. Cord blood samples for G6PD activity were actually available from 8139 patients. The remainder either had non-optimal cord specimens (clotted or insufficient) or were born outside our Hospital from whom cord bloods could not be taken.

Among the 8139 with cord blood samples successfully taken, the mean result for G6PD assay was 14.6 with a minimum value of 0.2 and a maximum value of 45.4. As we had a standard deviation of 3.28, 2 standard deviations from the mean (~2.5 percentile) give us a cut-off value of 8.05 (at 95% CI) below which defined a G6PD deficient neonate. It was well known that G6PD deficient variants were grouped into different classes corresponding with disease severity [1, 2].

Using a cut-off value of 8.05 Units/g Hemoglobin (U/g Hb), the overall incidence of G6PD deficiency was 3.13% of which 60% were males and only 40% were females. There was a significantly higher proportion of deficient males (*p* < 0.001) with significantly lower values as well (*p* < 0.001).

**Fig. 1:**
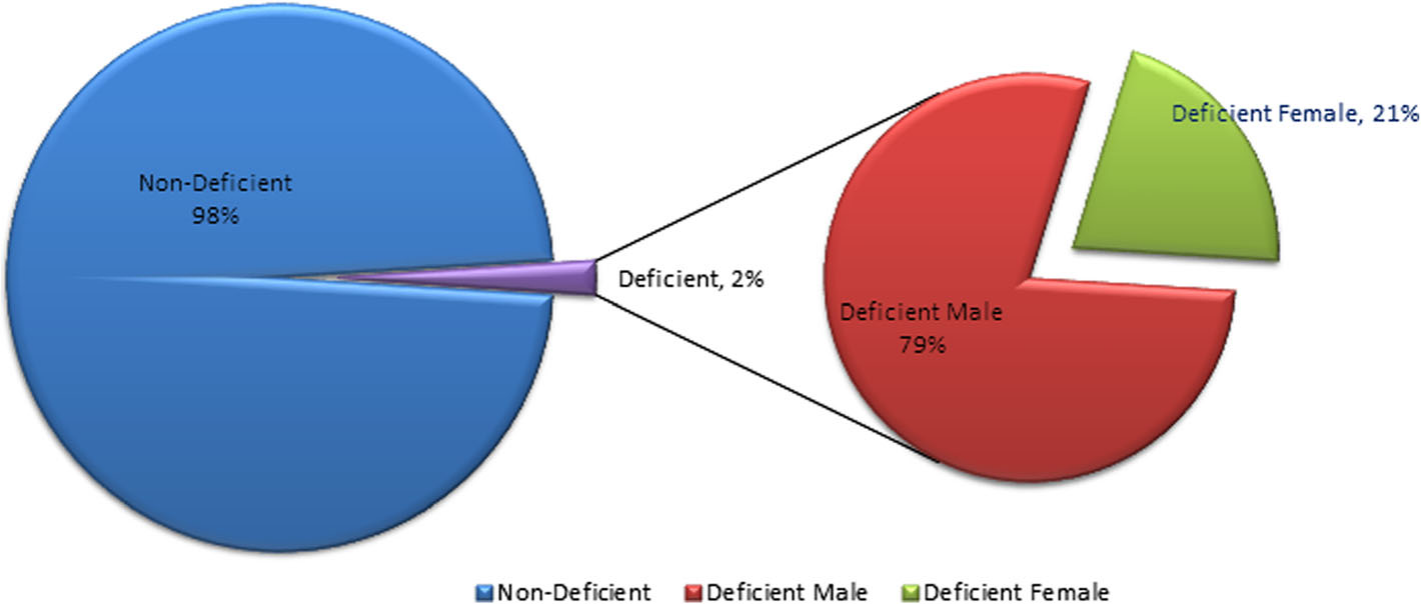
Incidence and Distribution of G6PD DEFICIENCY among neonates admitted in KAMC, during 2008 (*N* = 8139)

Presently however, when we applied using normal adult cut-off value of 5.7 U/g Hb, the overall incidence of G6PD deficiency was 2% of which 79% were males and only 21% were females (Fig. 1). There was a significantly higher proportion of deficient males (*p* < 0.001) with significantly lower values as well (*p* < 0.001).

We ran the analysis looking at gender differences in G6PD levels in both cut-off value of 5.7 and 8.05 U/g Hb for cord and peripheral blood samples and we found that there were equally highly significant differences (*p* < 0.001) in the levels between males and females in both samples (Table 2). Pearson chi-square test revealed a significantly higher proportion between gender groups of affected males (*p* = 0.001) consistent with an X-linked pattern of inheritance (M:F ratio) equal to 1.5–3.7:1 respectively. Among males alone, the computed incidence of the disease was 3.06% while female patients had an incidence of only 0.85% female neonates) with a male to female ratio of 3.7:1. The gender-specific incidence for males reinforces the universal screening practice also applied in our institution since it is within the WHO recommendation; that is, to screen for all neonates in populations with prevalence of ≥3% in males [2].

**Fig. 2:**
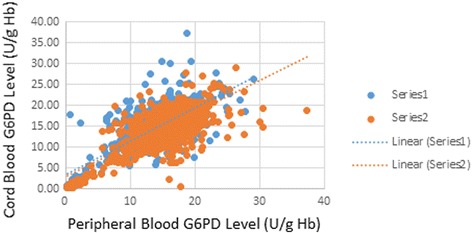
Concordance of G6PD Quantitative assay between Cord and Peripheral Blood Samples of KAMC neonates

To make a comparison between results obtained using cord blood and peripheral blood samples, we used the subset of patients from whom both samples were taken (Fig. 2, Table 1). A total of 1253 observations were analyzed. The mean result of G6PD activity assay was 14.52 (U/g Hb) for cord and 15.12 (U/g Hb) for peripheral blood samples. There was a significant difference between the mean results of the two samples (*p* = 0.0008). The G6PD activity assay was statistically lower in the cord than in the peripheral blood. This was consistent with the result of the concordance correlation coefficient of Lin with Rho c = 0.774 (*p* < 0.001, 95% CI: 0.752, 0.796) which only fell under the poor category (Pearson’s *p* = 0.78). However, when we dichotomized our patients into deficient and non-deficient, chi-square analysis revealed statistically comparable proportions of G6PD deficiency between the two samples using both cut-off points (*p* = 0.796 and *p* = 0.45 for cut-off 5.7 and 8.05, respectively). It was interesting to note that if we compared the mean G6PD values of the cord and peripheral blood samples in the deficient patients alone, no statistically relevant results were obtained using t-test (*p* = 0.3711) as opposed to the results obtained for the non-deficient group (*p* < 0.001, Table 1). Validity indices of cord blood G6PD assay, with peripheral blood as the gold standard, was confirmed as shown in Table 3.

## Discussion

The prevalence of G6PD deficiency is high in the Saudi Arabian population we think because of high consanguinity and the prevalence of endemic malaria. This justifies our thinking that neonatal screening is important for early diagnosis and identification of G6PD deficiency in infants before hospital discharge.

There are important observations that we have arrived at in this study. First, we note the preponderance of the disease in males which is congruent with an X-linked pattern of inheritance of G6PD deficiency. Moreover, the enzyme assays in the affected males are significantly lower than in the affected females which could explain the more severe nature of the disease in the male population.

Second, G6PD activity in the peripheral blood samples appears to be higher than in cord blood especially among non-deficient patients. This could reflect an up-regulation of G6PD activity as a response to oxidative stress in the newborn period by way of enhanced G6PD gene expression (i.e. transcription) [12–16]. Other possibilities are increased erythropoiesis resulting in normoblastemia, reticulocytosis, or possible other characteristics peculiar to fetal and neonatal erythrocyte metabolism [15, 17]. Cappellini and colleagues cite this as a diagnostic issue among neonates which could lead to false negative results [17]. This even raises doubts as to whether the peripheral neonatal blood is reflective of the true value or it could be a falsely elevated estimate. Therefore, we have used the 5.7 cut-off value as it is much more stringent and is based on the adult values. Using a much higher cut-off value would indeed show more number of G6PD deficient infants some of which could be false positive. Cut-off values used as described in the literature of <2.0 U/g Hb for profound and between 2 U/g to 7 U/g Hb for partial deficiency in the neonate may Indeed be too low.

Among the deficient group of neonates, regardless of the sample, they would have consistently low enzyme assays. We could only speculate that among this group of neonates, the same physiologic changes do not result in increased G6PD activity since the enzyme function is not optimal from the start. These observations reinforce the use of cord blood sample for G6PD deficiency screening among neonates since having a generally lower value than the peripheral blood samples, false negative results are minimized (i.e. negative predictive value of 99.5%, CI: 99.5%, 100%).

While we observed a statistically substantial discrepancy in the mean of G6PD assay between cord and peripheral blood samples using t-test, a chi-square analysis of the proportion of G6PD deficiency failed to show any meaningful difference. We also noticed that there were different normal G6PD level distributed among gender between cord blood and the peripheral blood samples.

This suggests that in so far as discriminating between deficient and non-deficient patients is concerned; there is no significant difference between the two groups. Again, this underscores the usefulness of cord blood in the universal neonatal G6PD deficiency screening.

## Conclusion

In this study, we found no difference between the cord and peripheral blood samples in discriminating between G6PD deficient and non-deficient neonates using quantitative enzyme activity assay. However, peripheral blood appears to have a higher G6PD quantitative assay than the cord blood. This reinforces the practice of using cord blood for neonatal screening since, having a generally lower value, the chances of missing a G6PD deficient neonate will be less (i.e. substantially low false negative results).

## Recommendation

The results of this study are best understood in the light of the following limitations. No randomization was carried out so the recommendations would not be as robust. The subset of patients for whom we analyzed correlation between the cord and peripheral blood for G6PD assay were mostly those for whom jaundice work-up was carried out, or those with borderline G6PD values in the cord blood, so that both cord and peripheral G6PD values had to be drawn. This may have imparted a sampling bias in this study.

An important question raised in this study which could be worth looking into in the future is establishing the relationship between cord and peripheral neonatal blood with that of older children. Should we be able to prove that the neonatal peripheral blood G6PD assay is indeed an overestimate of the true mature value? It might be plausible to set a higher cut-off point for labeling a newborn as G6PD deficient using a peripheral blood sample?

## Abbreviations

G6PD DEFICIENCY: glucose-6-phosphate dehydrogenase deficiency
G6PD: glucose-6-phosphate dehydrogenase
IRB: International Review Board
KAIMRC: King Abdullah International Medical Research Center
KAMC: King Abdulaziz Medical City
LIS: Laboratory Information System
MNGHA: Ministry of National Guard Health Affairs
MRN: medical record numbers
